# Middle Ear Exploration Results in Suspected Otosclerosis Cases: Are Ossicular and Footplate Area Anomalies Rare?

**Published:** 2013-06

**Authors:** Shadman Nemati, Naghavi Ebrahim, Ehsan Kazemnejad, Mohammad Aghajanpour, Omid Abdollahi

**Affiliations:** 1 *Otolaryngology-head and neck surgery Research Center, Amiralmomenin Hospital. Guilan University of Medical Sciences, Rasht, Iran.*; 2 *Department of biostatistics, Faculty of Midwifery and Nursing, , Guilan University of Medical Sciences, Rasht, Iran.*; 3 *Department of Otolaryngology-head and neck surgery, Guilan University of Medical Sciences, Rasht, Iran.*; 4 *Faculty of Medicine, Guilan University of Medical Sciences, Rasht, Iran.*

**Keywords:** Anomalies, Incus, Malleus, Middle ear exploration, Otosclerosis, Ossicular fixation, Stapes

## Abstract

**Introduction:**

Otosclerosis is a disease of bony labyrinth. Structural changes in the labyrinth often cause ossicular fixation, and thus conductive hearing loss. The purpose of this study was to evaluate middle ear exploration findings and frequency of ossicular and footplate area anomalies in patients with suspected otosclerosis referred to Amiralmomenin and Golsar Hospitals in Rasht, Iran.

**Materials and Methods:**

In 47 patients undergone middle ear exploration in Amiralmomenin and Golsar hospitals from April 2001 to March 2011, the intraoperative findings, and other data were extracted from the medical records of the patients. The data was analyzed using SPSS 17 software.

**Results:**

Frequency of fixation of stapes, malleus, and incus by age and sex in patients undergoing middle ear exploration showed that stapes had been fixed in 39 patients, malleus in 6 patients, and incus in 21 patients. Analysis of data showed that there was no significant association between sex and age with fixation of any of ossicles (P>0/05). Middle ear anomalies were seen in 16 cases (34.0%). Overhanging of facial nerve in 4 cases, thick stapedial crura in 5 cases, and perilymph gusher in 2 cases were the most frequent anomalies.

**Conclusion:**

This study show that the results of middle ear explorations in our patients in the north of Iran is somehow different from the typical otosclerotic cases, although the frequency of ossicular anomalies is better to be evaluated and compared in different areas of Iran, and other countries.

## Introduction

Otosclerosis (OS) is the most common etiology of conductive hearing loss in 15-50 years old patients with intact tympanic membrane (1). This is a disorder of bone which nearly exclusively involves the otic capsule (2).

The disease presents clinically in about 1% of Caucasians, and is transmitted as an autosomal dominant trait with incomplete penetrance. In practice otosclerosis is seen more often in women than men by the ratio of approximately 2:1 (3).

The prevalence of OS varies with race, and in whites the disease is found in 7.3% and 10.3% of temporal bones for men and women respectively. The stapes appears fixed in only 12.3% of patients with histopathologic evidence of otosclerosis (4).

Patients notice a slowly progressive hearing loss in one or both ears. With bilateral involvement one ear is usually affected more than the other. Tinnitus may be present, but vestibular dysfunction due to OS is rare. The differential diagnosis of conductive hearing loss with intact tympanic membrane includes: middle ear anomalies, ossicular chain disruptions, generalized disordes of bone metabolism such as Paget disorder, osteogenesis imperfecta and so on.

Besides fixations of stapes footplate, the head of the malleus may be fixed to the bony walls of the epitympanic recess (malleus head fixation): this may occur idiopathically, postoperatively or in the setting of tympanosclerosis (5).

The aim of our study was to analyze the findings of middle ear exploration, and the frequency of ossicular and footplate area anomalies in patients with suspected OS referred to Amir-Almomenin, and Golsar hospitals in Rasht, Guilan province of Iran April 2001 to March 2011.

## Materials and Methods

In a retrospective study of the operation results of 58 patients suspected to otosclerosis (OS) who underwent middle ear exploration for treatment of conductive hearing loss between 2001 to 2011 (Amir-Almomenin and Golsar hospitals, Rasht, north of Iran), the researchers studied patients` files and follow-up sheaths, and filled all questionnaires according to the records and documents related to intraoperative findings, medical, and audiological data. Those profiles with incomplete data, and the cases of exploration due to middle ear trauma, chronic otitis media, and revision otosclerosis were excluded from the study. The average patient with suspected OS, and a bone conduction level of 0-25 dB in the speech range, and an air conduction of 45-65 dB, was usually considered a suitable candidate for the surgery. Then the air bone gap was at least 15 dB, and the speech discrimination score was considered to be at least 60% for a good hearing improvement. All of the cases with type B or C tympanographs were excluded, and all of the surgery candidates had type A or As tympanographs. Nearly all of the cases underwent tunning fork tests with 256, 512, and 1024 Hertz diapasons, and the Rinne test would be negative at least with 256, and 512 Hz diapasons. When possible, in all of the cases stapedectomy or stapedotomy with Teflon piston insertion was performed.

Analysis of the data was performed by SPSS 17 software, and nonparametric chi-square and t-test were used to compare the findings. The level of significance was considered as 0.05.

## Results

A total of 47patients (between 18-61 years with a mean ± SD age of 36.45± 10.84 years) underwent middle ear exploration for suspected OS. Nearly 75% of patients were above 44 years old (Table 1). In 25 patients (53.2%) the right ear was operated, and in 21 cases (44.7%) the left ear, and in one patient (2.1%) both ears were operated. Nine cases (19.1%) had a positive personal history of ear trauma. Twenty two patients (46.8%) did not have any otologic or temporal bone trauma, and 16 patients (34%) did not remember any history of previous trauma to the ears.

**Table 1:** Demographic Data of Patients With Otosclerosis.

	No.	%	Mean age	SD
Male	13	27.66	34.31	13.05
Female	34	72.34	37.26	9.96
Total	47	100	36.45	10.84

(No= Number, SD= Standard Deviation)

All the 47 patients (100%) had hearing loss, and 24 cases (51.1%) had tinnitus. Episodic hearing loss was reported in only one patient; while 42 patients (89.4%) did not have any episodic hearing loss. 4 patients (8.5%) had a positive history of true vertigo. Two women (6%) had a positive history of hearing loss during pregnancy. 

Ten patients (21.2%) had a positive familial history of hearing loss. 20 patients (42.6%) did not have any history of hearing loss in their families, and the other 17 cases did not have any documented data about hearing loss in their families in their hospital admission files. Only one patient (2.1%) had a positive otological surgery in his family.

Otoscopic examinations of 42 cases (89.4%) were normal. Otoscopic findings of 4 patients (8.5%) were not mentioned in their history sheets. One patient had abnormal otoscopic findings. Tympanic membrane perforation was not seen in any of the cases. In 3 patients (6.4%) myringosclerotic plaques were reported. 11 patients had a wide external auditory canal (EAC). The diameter of EAC was normal in 17 cases, and it was not mentioned in the other 19 cases.

Audiograms with abnormal results were seen in 89.4% of patients. In the right ear the mean Air-bone gap was 24.45±18.94 dB, 19.68±16.06 dB, and 22.77±15.98 dB in the frequencies of 1000, 2000, 4000 HZ, respectively. In the left ear the mean Air-bone gap was 24.68±17.67 dB, 14.15±12.95 dB, and 18.83±15.51 dB in the frequencies of 1000, 2000 and 4000 Hz, respectively.

Carhartal notch was seen in 48/97 of audiograms. Sensorineural hearing loss [SNHL] was seen in 34% of patients under the age of 30, 12 cases, between the age of 30 and 40, 13 cases; and above the age of forty, 14 cases had a fixed stapes respectively. As a whole 39 cases had a fixed stapes. Stapes status was not mentioned in the operation sheet of 5 patients. 3 cases did not have a fixed stapes.

Six patients had a fixed malleus (four of them between 30 and 40 years old). Incus was fixed in 21 cases (under the age of 30, in seven cases; between 30 and 40 in four cases, and above 40, in ten cases, respectively). 

Malleus and incus status was not mentioned in the operation sheet of 38 and 21 cases, respectively. Table 2 shows the frequency of ossicular chain fixation in our patients according to sex. There was no statistical correlation between patients` age and sex with stapes, malleus and incus fixation (P>0/05).

Thick stapes crura was reported in 5 cases in them nearly always the posterior crus was involved. Stapedial footplate agenesis/ atresia was reported in 2 patients, and thick stapedial footplate was reported in one case. Overhanging facial nerve was mentioned in 4 patients' operation sheets. 

Perilymph gusher was reported in two cases. Ossicular chain dislocation was seen in one case, and ossicular chain agenesis was seen in two patients. 

There was no statistical correlation between patients` sex and age with the ossicular chain dislocation, stapes footplate thickness and other aforementioned anomalies (P>0/05).

**Table 2:** The Frequency of Ossicular Chain Fixation in Patients With Otosclerosis.

		** Stapes**			**Malleus**			**Incus**		
		**+**	**-**	Undet.	**+**	**-**	Undet**.**	**+**	**-**	Undet**.**
**Male**	**No.**	11	1	1	2	1	10	5	2	6
	**%**	84.6	7.7	7.7	15.4	7.7	76.9	53.8	15.4	46.2
**Fem**	**No.**	28	2	2	4	2	28	16	3	15
	**%**	82.3	5.9	5.9	11.8	5.9	82.4	47.1	8.8	44.1
**Total**	**No.**	39	3	3	6	3	38	21	5	21
	**%**	83	6.4	6.4	12.8	6.4	80.9	44.7	10.6	44.7

According to Sex; (No.= Number, Undet.= Undetermined)

## Discussion

The aim of our study was to evaluate middle ear exploration findings, and frequency of ossicular and footplate area anomalies in patients with suspected otosclerosis (OS) referred to Amiralmomenin and Golsar Hospitals in Rasht, in north-west of Iran between 2001 and 2011. The clinical incidence of OS is more frequent in females with an approximately 2:1 female to male ratio (6); in our study a ratio of 2.6:1 was obtained.

There is usually a positive familial history of hearing loss, and in our study 21.2% of cases had a positive familial history of hearing loss, compared to other study in Tabriz, Iran in which 16.6% of 30 OS cases had positive familial history (7). Tinnitus is a relatively common complaint in OS, and may be an indicator of sensorineural degeneration, and patients rarely have complaints of vertigo (5); in our study 51.1% of cases had tinnitus, and 8.5% of patients had a positive history of vertigo. 

Emery et al. reviewed 25 patients with congenital fixed stapes, who underwent stapedectomy and found that the mean air-bone gap was 10-20 dB (8), and there was no correlation between the patients` age and ossicular chain anomalies, and this was correlated with our study. Albert and colleagues reported that the most common isolated congenital ossicular anomalies are stapedial ankylosis, and incustapedial discontinuity (9); and in our study this finding had been approved. Wetanabe et al. reported a bilateral congenital agenesis of incus long process (10), and in our study only two patients had ossicular chain abnormality. 

Ossicular chain anomalies are rare; and generally, the results of otologic surgeries are suboptimal in these cases (12). The incidence of obliterative OS according to the literature varies from 4% to 31% (11,12). In the study of Vincent R. et al (12) the incidence of obliterative OS was 14.7% in their children series (5/34 cases), and 2.6% in the elderly series (9/340 cases). In our cases, stapedial footplate agenesis/ atresia was reported in 2 patients, and thick stapedial footplate was reported in one case. 

As we searched in the Persian and English literature, we could not find any similar study in which the researchers investigated the frequency of ossicular and footplate area anomalies in patients with suspected OS. Many of the papers are limited case reports, and it seems that the frequency of ossicular anomalies is better to be evaluated and compared in different areas of Iran and other countries. There was a noticeable proportion of our patients whiched showed to have anomalies of footplate area and stapes, and although we have no data of other parts of Iran and other countries to compare these findings, but this can teach us, and especially our residents and junior otosurgens the importance of preoperation imaging studies in these patients. 

## Conclusion

This study demonstrated that middle ear exploration findings in our survey in the north of Iran (Guillan province) are not identical to other patients with typical otosclerotic in other studies. The prevalence of ossicular chain abnormalities, especially in the foot plate region seems to be high in our patients; thus, comparing these findings with other researches in other countries is recommended.
